# Unraveling the Contribution of Fluid Therapy to the Development of Augmented Renal Clearance in a Piglet Model

**DOI:** 10.3389/fphar.2020.607101

**Published:** 2021-01-26

**Authors:** Laura Dhondt, Siska Croubels, Peter De Paepe, Klara Goethals, Pieter De Cock, Mathias Devreese

**Affiliations:** ^1^ Department of Pharmacology, Toxicology and Biochemistry, Faculty of Veterinary Medicine, Ghent University, Merelbeke, Belgium; ^2^ Heymans Institute of Pharmacology, Faculty of Medicine and Health Sciences, Ghent University, Ghent, Belgium; ^3^ Department of Nutrition, Genetics and Ethology, Faculty of Veterinary Medicine, Ghent University, Merelbeke, Belgium; ^4^ Department of Pharmacy, Ghent University Hospital, Ghent, Belgium; ^5^ Department of Paediatric Intensive Care, Ghent University Hospital, Ghent, Belgium

**Keywords:** augmented renal clearance, fluid therapy, large animal model, piglet, iohexol, para-aminohippuric acid, amikacin

## Abstract

Augmented renal clearance (ARC) observed in the critically ill pediatric population has received an increased attention over the last years due to its major impact on the disposition and pharmacokinetics of mainly renally excreted drugs. Apart from an important inflammatory trigger, fluid administration has been suggested to contribute to the development of ARC. Therefore, the primary objective of this study was to evaluate the effect of continuous intravenous fluid administration on renal function using a conventional piglet animal model and to quantify the impact of fluid administration on the pharmacokinetics of renally excreted drugs. At baseline, twenty-four piglets (12 treatment/12 control; 7 weeks old, all ♂) received the marker drugs iohexol (64.7 mg/kg body weight (BW)) and para-aminohippuric acid (10 mg/kg BW) to quantify glomerular filtration rate and effective renal plasma flow, respectively. In addition, the hydrophilic antibiotic amikacin (7.5 mg/kg BW) was administered. Following this baseline measurement, the treatment group received fluid therapy as a constant rate infusion of 0.9% saline at 6 mL/kg/h over 36 h. After 24 h of fluid administration, the marker drugs and amikacin were administered again. When comparing both groups, a significant effect of fluid administration on the total body clearances of iohexol (*p* = 0.032) and amikacin (*p* = 0.0014) was observed. Clearances of iohexol and amikacin increased with on average 15 and 14%, although large interindividual variability was observed. This led to decreased systemic exposure to amikacin, which was manifested as decrease in area under the plasma concentration-time curve from time 0 h to infinity from 34,807 to 30,804 ng.h/mL. These results suggest that fluid therapy is a key factor involved in the development of ARC and should be taken into account when administering mainly renally excreted drugs. However, further research is necessary to confirm these results in children.

## Introduction

Intravenous (IV) fluids are often administered to hospitalized pediatric patients. Generally, fluid therapy is provided either to replace ongoing losses of water and electrolytes under normal physiological conditions, also known as maintenance therapy, or to correct for any existing electrolyte and water deficits, known as fluid resuscitation (Sterns, 2019). Guidelines are available to guide the volume and type of administered fluid; however they are not always unequivocal and remain subject of extensive debate (Perel et al., 2013; Lira and Pinsky, 2014; Padua et al., 2015; Feld et al., 2018; National Institute for Health and Care Excellence, 2020). IV fluid administration is undoubtedly no exact science. Determining the optimal volume, administration rate, and composition of IV fluids can be a difficult task. Consequently, errors in prescribing fluid therapy are not uncommon.

Detrimental effects have been associated with both excessive and too limited IV fluid administration (Hilton et al., 2008; Hopf and Morrissey, 2019). Too much fluid is associated with complications such as tissue edema and poor cardiac function, whereas too little fluid may lead to inadequate tissue perfusion and impaired wound healing (Hopf and Morrissey, 2019). Furthermore, the administration of IV fluids is thought to contribute to the development of augmented renal clearance (ARC) (Udy et al., 2010). ARC refers to a supraphysiological state of the kidney and is characterized by an increased elimination of circulating solutes compared to normal baseline (De Waele et al., 2015; Wu et al., 2016). This is particularly relevant for critically ill patients, where ARC may lead to subtherapeutic plasma concentrations of mainly renally excreted drugs, such as aminoglycosides and ß-lactam antibiotics. Consequently, ARC may negatively affect the patient's clinical outcome. ARC is a well described phenomenon in the critically ill adult patient population but evidence on its occurrence in critically ill children is accumulating as well (De Cock et al., 2015; Van der Heggen et al., 2019). A recent study of Van Der Heggen *et al.* demonstrated a high prevalence of ARC (67%) in critically ill children (Van der Heggen et al., 2019). Despite the hypothesis that fluid therapy can contribute to ARC, the volume of administered fluid is not consistently identified as risk factor for development of ARC (Udy et al., 2010; Campassi et al., 2014; Declercq et al., 2016; Hirai et al., 2016; Cook and Hatton-Kolpek, 2019; Van der Heggen et al., 2019). Furthermore, limited data are available on the effect of continuous IV fluid administration on the renal function. Leake *et al.* observed in infants weighing less than 2 kg a higher inulin clearance when infusing fluid at a high rate (10.3 mL/kg/h) compared to a low rate (3.6 mL/kg/h). Conversely, in children weighing more than 2 kg, inulin clearance did not significantly increase when administering fluid at hight rate (Leake et al., 1976). Also, inconsistent results about the glomerular response to IV bolus administered saline are reported in humans (Crawford and Ludemann, 1951a; Stenvinkel et al., 1992; Hahn et al., 2016).

Due to their resemblance with humans with respect to size, anatomy, and physiology of the kidney and other organ systems, pigs could be an appropriate animal model to study the underlying mechanism of ARC, the contribution of fluid administration to the development of ARC, and the effect of ARC on the pharmacokinetics (PK) of renally excreted antimicrobials (Gasthuys et al., 2016). Previously, Gasthuys *et al.* demonstrated that the maturation of the glomerular filtration rate (GFR) was comparable in humans and conventional pigs, making the piglet a potential good preclinical model for pediatric drug research (Gasthuys et al., 2017a). Furthermore, we recently demonstrated that mature values of GFR, effective renal plasma flow (ERPF), anion secretion, and tubular reabsorption were in the same order in humans and conventional pigs (Dhondt et al., 2020).

The primary aim of the current study is to investigate the effect of a constant rate infusion (CRI) of 0.9% saline on glomerular filtration and renal plasma flow, using the conventional pig as animal model. Due to the high prevalence of ARC in the pediatric population, it was decided to perform this study in juvenile pigs (Udy et al., 2014; De Waele et al., 2015; Mulder et al., 2019; Van der Heggen et al., 2019). The fluid rate was chosen as such that the fluid requirements of piglets were moderately overestimated, mimicking a condition of moderate extensive fluid administration in children. The renal function was evaluated using iohexol and para-aminohippuric acid as markers. Furthermore, it was evaluated if fluid administration alters the PK of renally cleared drugs using amikacin as a test compound. In addition, it was investigated whether amikacin clearance could serve as an estimate for the GFR.

## Materials and Methods

### Animals

The current study was conducted with consent of the Ethical Committee of the Faculty of Veterinary Medicine and the Faculty of Bioscience Engineering of Ghent University (EC 2017/24). Care and use of animals were in full compliance with the Belgian (Anonymous, 2013) and European legislation on animal welfare and ethics (2010/63/EU) (The European Parliament and the Council of the European Union, 2010; Anonymous, 2013).

Twenty-four clinically healthy, 6-week-old male piglets (Landrace x Large White, Seghers Hybrid®, RA-SE Genetics, Belgium) were recruited for this study. Due to practical issues, the trial was divided over three different time periods spread over three months. Piglets were equally handled over these periods. In each period, eight piglets were randomly divided over treatment and control group. Upon arrival, piglets were group-housed (4 piglets per pen) in standard pig pens (2.30 × 2.40 m) with *ad libitum* access to water and feed (Piggistart Opti®, Aveve, Leuven, Belgium). During the entire experimental period, stables were enriched with rubber toys, balls of different size, and cotton towels. After a 5-day acclimatization period, a double-lumen jugular catheter was surgically inserted following the procedure described by Gasthuys *et al.*, permitting IV infusion and blood collection (Gasthuys et al., 2017b). To allow urine collection, a human stoma ring (Esteem synergy® Uro, 48 mm, ConvaTec, Braine-l’Alleud, Belgium) was attached around the prepuce of the piglets during anesthesia (Gasthuys et al., 2017b). After surgery, the piglets were housed individually to avoid displacement of the catheters and stoma rings. Catheters were flushed twice daily with heparinized 0.9% saline (50 IU/mL) and the bandages were changed daily. Natural light was provided by translucent windows. The mean (± standard deviation (SD)) stable temperature was 24.7 ± 0.99 °C. Body temperature was measured twice daily using a Biothermo Lifechip (Allflex, Vitré, France), placed in the *musculus gluteus maximus* during surgery. All piglets were weighed prior to surgery and the day before drug or fluid administration.

### Trial Design

**FIGURE 1:**
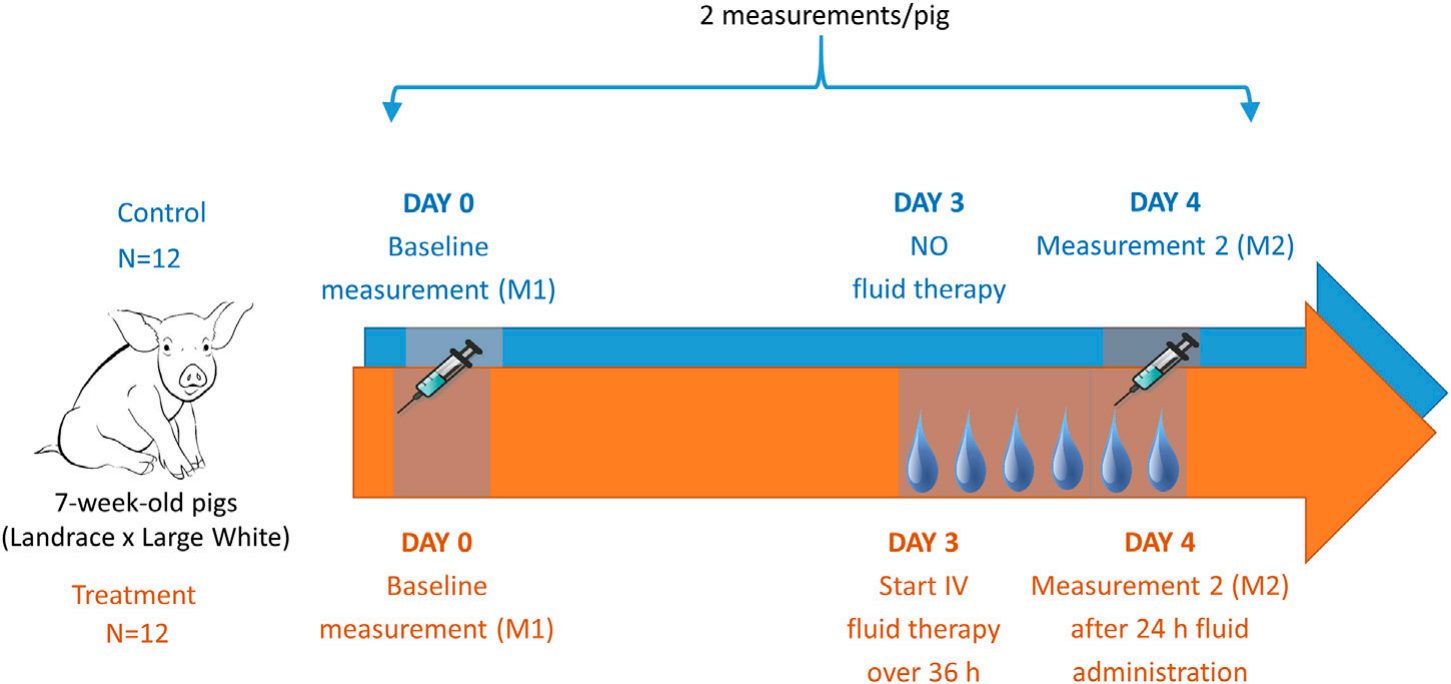
Graphical illustration of the trial design including in total 24 piglets. After placement of a jugular catheter, baseline pharmacokinetics (PK) were determined of iohexol (64.7 mg/kg BW), PAH (10 mg/kg BW), and amikacin (7.5 mg/kg BW). After a two-day rest, fluid at a rate of 6 mL/kg/h of 0.9% saline was administered over 36 h to 12 piglets (treatment group). After 24 h of fluid administration, the PK determination of the above mentioned drugs was repeated in both the fluid treated (n = 12) and the control (n = 12) piglets.

A graphical illustration of the conducted trial is shown in Figure 1. The piglets were allowed to recover for one day after surgery. The following day (Day 0), baseline measurements were performed (measurement 1 or M1). All piglets (7 weeks old, 14.1 ± 1.6 kg body weight (BW), fasted for 10 h) received single, consecutive IV boluses of iohexol [64.7 mg/kg BW, Omnipaque 300® (GE Healthcare, Eindhoven, The Netherlands)], para-aminohippuric acid (PAH, 10 mg/kg BW), and amikacin (7.5 mg per kg BW, Amukin® 1g/4 mL, S.A. Bristol-Myers Squibb, Brussels, Belgium) using the proximal lumen of the jugular catheter. The commercially available analytical standard of sodium PAH, purchased from Sigma-Aldrich (Bornem, Belgium), was prior to administration dissolved in 0.9% saline solution at a final concentration of 100 mg/mL PAH. After administration, venous blood samples (1.5 mL) were collected from the distal lumen of the catheter into K_3_-EDTA collection tubes (Vacutest Kima®, Piove di Sacco, Italy) at 0 (prior to administration), 5, 10, 20, 30, 45, and 60 min and 1.5, 2, 4, 6, 8, and 10 h after administration (p.a.) and immediately stored on ice. Samples were centrifuged (2,095 x g, 10 min, 4 °C) within 2 h and aliquots of plasma were stored at ≤ −80 °C until analysis. Urine bags were emptied regularly. At each collection point, the collected volume was noted and an aliquot for analysis was taken. Urine samples were stored at ≤−15 °C. Since inflammation is considered a major trigger of ARC, a total white blood cell count and formula were performed before administration of the drugs to rule out the presence of inflammation and/or infection. These measurements were performed by Medvet BVBA clinical laboratory (Antwerp, Belgium). Piglets demonstrating an extreme deviation in hematology values in combination with an elevation in body temperature were excluded from the study. During this baseline measurement all piglets received *ad libitum* water. Feed was provided 4 h p. a. Thereafter, the piglets could recover for 2 days.

The third day after the baseline measurement (Day 3), fluid administration was started in twelve piglets (treatment group). The piglets received 0.9% saline as a CRI at 6 mL/kg/h for 36 h, administered via the proximal lumen of the catheter. In contrast to the untreated piglets (control group), to which *ad libitum* water was provided, these piglets had no additional access to water. Twenty-four hours after the start of the infusion, the same drugs as during the baseline measurement were administered to the animals (16.1 ± 1.8 kg, fasted for 10 h) (M2). Blood sampling and sample handling were performed the same way as described above. Again, an additional sample was taken for total white blood cell count and formula. Before and after 12, 24, and 36 h of fluid administration a blood sample was taken to assess hematocrit levels. Hematocrit was measured by Medvet BVBA (Antwerp, Belgium).

### Bioanalysis Method of Iohexol, Para-Aminohippuric Acid, and Amikacin in Plasma and of Para-Aminohippuric Acid in Urine

Total plasma iohexol and PAH concentrations were quantified simultaneously using an ultra-high performance liquid chromatography-tandem mass spectrometry (UHPLC-MS/MS) method previously described by Dhondt et al. (2019) with minor modifications (Dhondt et al., 2019). More specifically, a ^13^C-labeled IS for PAH analysis (^13^C_6_-PAH, Alsachim, Illkirch Graffenstaden, France) was employed instead of para-aminobenzoic acid. The lower limit of quantification (LLOQ) was 0.25 μg/mL for both compounds. The same UHPLC method was used for analysis of PAH in urine samples. Information about the applied sample preparation as well as validation results are presented in the Supplementary Material. The LLOQ of PAH quantification in urine was 0.25 μg/mL.

Plasma concentrations of amikacin were quantified using an in-house validated UHPLC-MS/MS method. The LLOQ was set at 0.50 μg/mL. A broad method description and validation results are provided in the Supplementary Material section.

### Pharmacokinetic Modeling

Pharmacokinetic modeling of the plasma concentration-time data was performed using Phoenix® 8.1 (Certara, USA). Values below the LLOQ were excluded from the dataset. This resulted in percentages of extrapolated area under the curve (AUC) of less than 10% for each compound. The PK models were fit to each individual concentration-time profile. The structural model for amikacin was a two-compartmental model with first-order elimination. A multiplicative error model was used. For iohexol and PAH, the plasma concentration-time data was best described by a two-compartmental model with first-order elimination and a multiplicative residual error model. However, in six out of 48 iohexol PK measurements (from five different piglets) and nine out of 48 PAH PK measurements (from eight different pigs), a two-compartmental model with multiplicative error model failed to describe the data appropriately. This resulted in an unacceptable precision of the individual PK parameter estimations, as reported by the coefficient of variation (CV%). For those measurements, the profile was better described by a one-compartmental model with first-order elimination, which resulted in acceptable CV%. In those cases, a multiplicative error model was used for iohexol, whereas an additive error model was used in case of PAH. The estimated primary parameters were volume of distribution (V_d_) and total body clearance (CL_TOT_). Also the following secondary variables were calculated: volume of distribution at steady state (V_ss_) and the area under the curve from time 0 h to infinity (AUC_0→inf_).

The cumulative amount of unchanged compound recovered in the urine (A_e_) was calculated taking the sum of the amount excreted at every collection point. This amount was calculated by multiplying the observed concentration by the volume collected at every collection point. Subsequently, the renal clearance (CL_R_) of PAH, which was used to evaluate the ERPF, was calculated by following formula:

$$CL_{R}=\frac{A_{e}}{AUC_{0\to inf}}$$

The total body clearance of iohexol was used for the determination of the GFR.

The percentage change in plasma volume was calculated from the hematocrit value under the assumption of constant red cell volume using the formula of van Beaumont (van Beaumont, 1972):

$$\text{\%}\Delta \text{P}=\frac{100}{100-H_{1}}\;\times \;\frac{100\left(H_{1}-H_{2}\right)}{H_{2}}\text{\%}$$

with H_1_ the hematocrit value before fluid administration and H_2_ the hematocrit value at 12, 24, or 36 h after the start of the fluid administration.

### Statistical Analysis

Statistical analyses were performed using Rstudio version 3.6.1 and Prism GraphPad version 6.01. To evaluate if enhanced clearances were present at M2 within each group, a one-sided Wilcoxon signed rank test was performed. A one-sided Mann-Whitney *U* test was used, to assess if the increases in clearance were more pronounced in the treatment than in the control group. To evaluate the effect of fluid administration on V_ss_, AUC_0→inf_ and urine output two-sided tests were performed. Changes in body temperature and white blood cell count within each group were evaluated using a two-sided Wilcoxon signed rank test, whereas differences in change in body temperature between groups were assessed using a Mann-Whitney *U* test. The level of significance was set at 0.05. Median (25th percentile-75th percentile) is presented.

Correlations were determined using a Pearson correlation test. To assess the agreement between two measurements in the control group, Bland-Altman plots were plotted. To evaluate changes in hematocrit over time a linear regression analysis was performed. In order to evaluate differences in plasma volume changes, a general linear model with time as within-subject factor and group as between-subject factor was used. To evaluate changes in plasma volume between groups at every time point, a Mann-Whitney *U* test was performed.

Coefficients of variation (CV%) were calculated for repeated measurements by dividing the standard deviation by the mean of the measurements. The reproducibility (R) of the clearance measurements was calculated as a mean coefficient of variation as the square root of

$$\overset{n}{\underset{i=1}{\sum }}\frac{(CV_{i}{)}^{2}}{n}$$

where CV_i_ is the coefficient of variation in subject *i* for duplicate determinations and n is the number of subjects (Gaspari et al., 1998). The calculated reproducibility in this study accounted for both methodological and biological variations.

## Results

### Hematology and Body Temperature

**FIGURE 2:**
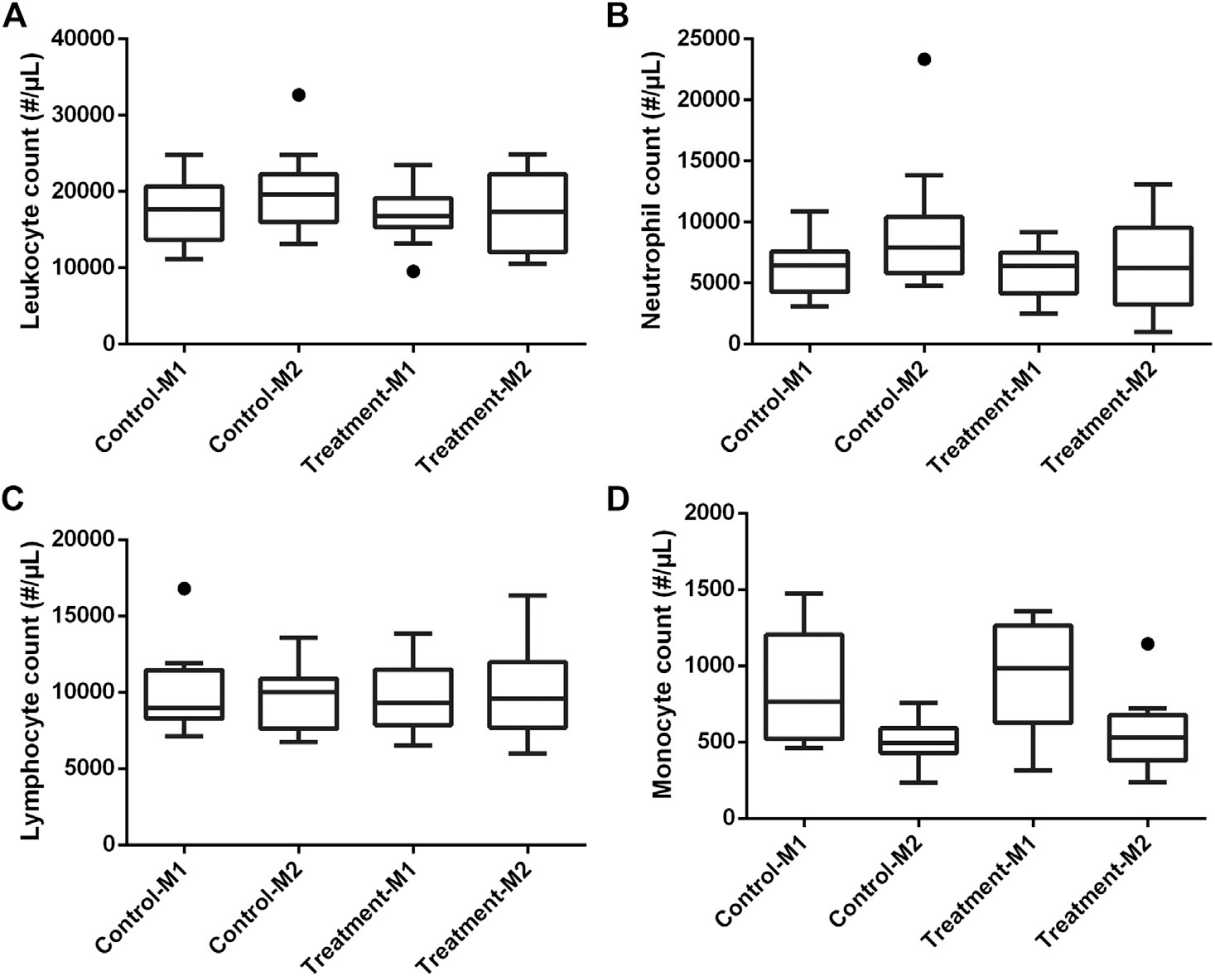
Boxplots of leukocyte **(A)**, neutrophil **(B)**, lymphocyte **(C),** and monocyte **(D)** count in the control (n = 12) and treatment (n = 12) group determined just before baseline measurement (M1) and before the second administration of drugs (M2). The boxplots illustrate the median and 25th and 75th percentiles. The whiskers extent from the 25th/75th percentile to the minimum/maximum value, respectively, no further than 1.5 times the interquartile range. Outliers, defined as a value exceeding 1.5 the interquartile range, are plotted individually. The deviating values in leukocyte and neutrophil count of the excluded piglet are clearly illustrated as outliers.

**FIGURE 3:**
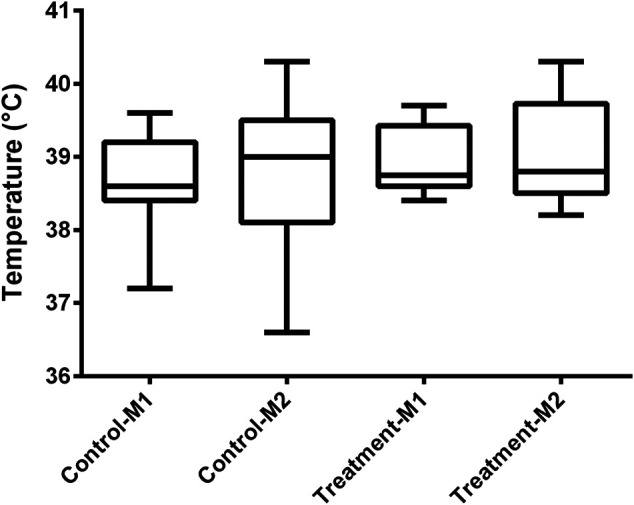
Boxplot of the body temperature in the control (n = 11) and treatment (n = 12) group determined just before baseline measurement (M1) and before the second administration of drugs (M2). The boxplots illustrate the median and 25th and 75th percentiles. The whiskers extent from the 25th/75th percentile to the minimum/maximum value, respectively, no further than 1.5 times the interquartile range. Outliers, defined as a value exceeding 1.5 the interquartile range, are plotted individually.

In all piglets, a double lumen jugular catheter was inserted successfully. These were functional throughout the whole study period. During the trial, the piglets showed normal activity and appetite. No adverse effects were observed during the constant rate infusion of 0.9% saline. Figure 2 shows the leukocyte (A), neutrophil (B), lymphocyte (C), and monocyte (D) count in the control and treatment group determined just before baseline measurement (M1) and before the second administration of drugs (M2). In one piglet (control group, third period), a substantial elevated leukocyte count (32,690/µL) against normal reference range (11,300–22,800/µL) was observed around the time of second administration of the drugs. This was mainly attributed to an increase in neutrophil count (23,341/µL, normal reference range: 3,100–9,600/µL) as shown in Figure 2B. Simultaneously, an increase of 1.2 °C in body temperature against the baseline measurement was observed. Therefore, it was decided to exclude this piglet in the further calculations. After exclusion of this piglet, no significant differences in leukocyte (control: *p* = 0.29; treatment: *p* = 0.39), neutrophil (control: *p* = 0.08; treatment: *p* = 0.39), and lymphocyte count (control: *p* = 0.59; treatment: *p* = 0.75) were observed between the first and second measurement within each group. Monocyte counts were in both groups significantly decreased at the second evaluation (control: *p* = 0.03; treatment: *p* = 0.02; Figure 2D). Nevertheless, 87% of the values were within the reference interval (300–1,200/µL). Also, the outlier in monocyte count (1,144/µL), observed at second measurement in the treatment group, was still within this reference interval (Figure 2D). An outlier in lymphocyte count was observed in a control piglet at first measurement (Figure 2C). Nevertheless, this increased lymphocyte count was not accompanied by a deviation in body temperature (39.2 °C), nor abnormal behavior. Therefore, this piglet was not excluded from the dataset. In Figure 3, boxplots of the body temperature measured before the administration of drugs in the control (n = 11) and treatment group (n = 12) are shown. No statistically significant differences in body temperature were observed between the two measurements within the control (*p* = 0.66) and treatment group (*p* = 0.91) and between groups (*p* = 0.69).

### Effect of Fluid Administration on PK Parameters of Iohexol, Para-Aminohippuric Acid, and Amikacin

**FIGURE 4:**
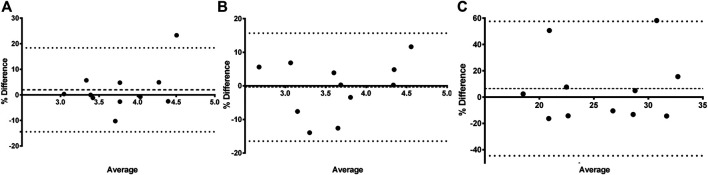
Bland-Altman plot of the difference between iohexol **(A)** total body clearance measurement 1 and measurement 2 vs. the mean of the two measurements evaluated within the control group (n = 11). The difference is expressed as percentages of the values on the axis [(measurement 2—measurement 1)/mean]. The mean bias is presented as a dashed line. The 95% lower limit of agreement (LLA) and upper limit of agreement (ULA) are presented by dotted lines. Similar plots were constructed for amikacin **(B)** and para-aminohippuric acid **(C)**.

In Table 1 the PK parameter estimates of iohexol, PAH, and amikacin are presented. For all three compounds, a significant effect of fluid administration on the total body clearance of the drugs was observed within the treatment group. For iohexol, an average increase of 15% (range: −5 to + 45%) in total body clearance was observed after fluid administration, whereas in the control group the average increase was limited to 2% (range: −10 to +26%). The latter highlights the reproducibility of the iohexol measurement (R = 5.81%) in piglets (Table 2). In the control group, repeated measurements of GFR with iohexol resulted in individual differences of less than 10% between the two measurements in 10 out of 11 piglets, as illustrated in Figure 4A. In one control piglet an unexpected increase in iohexol clearance was observed. A possible explanation could be an upcoming infection since a transient elevation in body temperature up to 41 °C was observed at 12 h after the start of the fluid administration. It was decided to not exclude this piglet out of the data set, since no major deviations in hematology were observed. Nevertheless, statistical analysis revealed that the increase in iohexol clearance was significantly higher in the treated group than in the control group (*p* = 0.032). Furthermore, the effect of fluid administration on the clearance of iohexol showed large interindividual differences. In five out of 12 piglets iohexol clearance during fluid administration was within −10 to + 10% of the baseline measurement. In respectively four and one out of 12 piglets an increase of 10 to 20% and 20 to 30% against baseline was observed. In two other piglets the increase exceeded 30%, namely 35 and 45%.

**TABLE 1:** Pharmacokinetic parameters of iohexol (64.7 mg/kg BW), para-aminohippuric acid (PAH, 10 mg/kg BW), and amikacin (7.5 mg/kg BW) after single intravenous bolus administrations to 7-week-old piglets. In the control group, the baseline and second measurements were performed under identical conditions. In the treatment group, the second measurement was performed after 24 h fluid administration consisting of 0.9% saline at 6 mL/kg/h constant rate infusion. The median (25^th^‐75^th^ percentile) is represented.

IOHEXOL				
	Control group (n = 11)		Treatment group (n = 12)	
	**Baseline measurement**	**Measurement 2**	**Baseline measurement**	**Measurement 2**
CL_TOT_ (mL/min/kg)^*^	3.82 (3.41–4.01)	3.72 (3.41–4.18)	3.55 (3.34–4.33)^†^	4.25 (3.88–4.55)
V_ss_ (mL/kg)^*^	371.65 (366.86–390.63)	383.76 (363.69–392.63)	389.34 (361.79–405.96)^†^	412.17 (386.92–444.70)
AUC_0→inf_ (ng.h/mL)	282,347 (268,682–315,970)	289,572 (258,373–315,804)	303,801 (249,314–322,605)^†^	253,727 (236,903–277,969)
**PAH**				
	**Control group (n = 11)**		**Treatment group (n = 12)**	
	**Baseline measurement**	**Measurement 2**	**Baseline measurement**	**Measurement 2**
CL_TOT_ (mL/min/kg)	24.25 (21.72–29.15)	26.22 (22.19–29.45)	24.38 (21.37–27.90)^†^	25.37 (24.54–31.79)
CL_R_ (mL/min/kg)^Δ^	9.62 (8.01–11.32)	12.55 (11.53–15.94)	11.46 (9.60–13.17)	15.53 (13.07–18.26)
V_ss_ (mL/kg)	486.18 (445.81–534.54)	493.82 (409.79–566.10)	486.43 (421.64–556.04)^†^	577.24 (530.76–628.57)
AUC_0→ inf_ (ng.h/mL)	6,874 (5,725–7,673)	6,357 (5,660–7,530)	6,843 (5,982–7,808)	6,572 (5,243–6,793)
**AMIKACIN**				
	**Control group (n = 11)**		**Treatment group (n = 12)**	
	**Baseline measurement**	**Measurement 2**	**Baseline measurement**	**Measurement 2**
CL_TOT_ (mL/min/kg)^*^	3.68 (3.40–4.06)	3.67 (3.12–4.04)	3.59 (3.29–3.98)^†^	4.06 (3.82–4.36)
V_ss_ (mL/kg)	510.76 (506.30–521.05)^†^	482.45 (432.55–506.83)	523.10 (497.01–565.16)	539.22 (489.56–551.58)
AUC_0→inf_ (ng.h/mL)^*^	34,000 (30,839–36,830)	34,076 (31,108–40,373)	34,807 (31,420–38,071)^†^	30,804 (28,674–32,763)

CL_TOT_: total body clearance; CL_R_: renal clearance; V_ss_: volume of distribution at steady state; AUC_0→inf_: area under the plasma concentration-time curve from time 0 h to infinity. If no significant differences were present, no annotations were made.

Significant differences (*p* < 0.05) in PK parameter between baseline measurement and measurement 2 within each group are annotated with ^†^.

Significant differences in change of a PK parameter (M2-M1) between treatment and control group are indicated with *.

^Δ^ PAH renal clearances were assessed in four pigs per group.

**TABLE 2:** Reproducibility of the total body clearance of iohexol, para-aminohippuric acid (PAH), and amikacin after administration of iohexol (64.7 mg/kg BW), PAH (10 mg/kg), and amikacin (7.5 mg/kg BW) to 7-week-old piglets with *ad libitum* access to water (control group, n = 11)).

Parameter	Iohexol	Amikacin	PAH
N	11	11	11
Mean CV (%)	3.64	4.52	13.36
Range of CV (%)	0 to 16	0 to 10	2 to 41
R%	5.81	5.52	18.14

**FIGURE 5:**
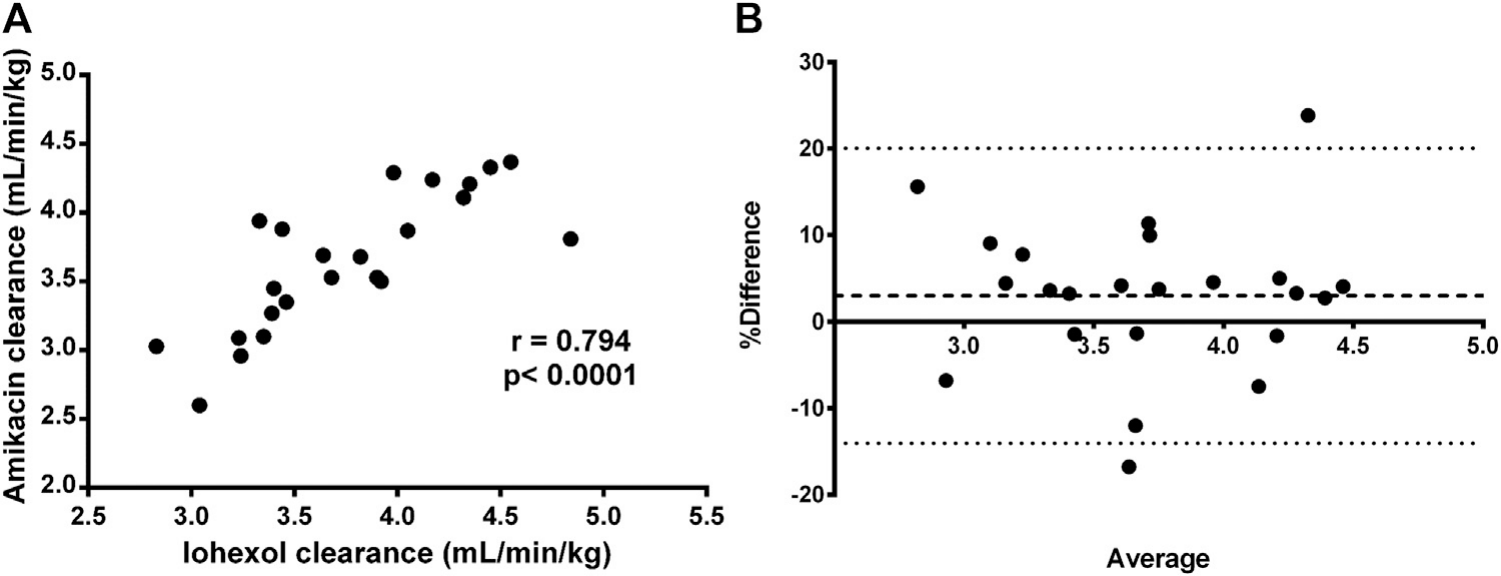
Graphical correlation between iohexol (*x*-axis) and amikacin (*y*-axis) clearance **(A)** and Bland-Altman plot of the difference **(B)** between iohexol and amikacin total body clearance, expressed as percentages of the values on the axis [(CL_IOH_–CL_AMI_)/mean)], vs. the mean of the two clearances, after administration of iohexol (64.7 mg/kg BW) and amikacin (7.5 mg/kg BW) to 7-week-old piglets with *ad libitum* access to water. These plots were constructed by using only the baseline measurements (measurement 1) of each group (n = 23). The mean bias is presented as a dashed line. The 95% lower limit of agreement (LLA) and upper limit of agreement (ULA) are presented by dotted lines.

Similar results were observed for amikacin. The total body clearance in the treatment group increased after fluid administration with on average 14% (range: −5 to +33%). In contrast, the change in total clearance in the control group remained limited to −0.06% (range: −13% to 12%). As a consequence, a good reproducibility (R = 5.52%) of the amikacin clearance was observed, as illustrated in Table 2 and Figure 4B. When comparing the control and treated group, a statistically significant higher increase in amikacin clearance was observed after fluid administration (*p* = 0.0014). Again, interindividual differences were present. In five out of 12 piglets of the treatment group, total amikacin clearance values during fluid administration increased less than 10% against baseline measurement. In the +10–+20% and +20–+30% interval each time three piglets were present. One piglet showed an increase in amikacin clearance of more than 30% after fluid administration. When comparing baseline total iohexol and amikacin clearances a reasonably good correlation (r = 0.794; *p* < 0.0001; n = 23) and agreement was observed, as illustrated in Figure 5. Also under perturbed, fluid administration conditions, a good correlation between iohexol and amikacin clearances was observed (r = 0.765; *p* = 0.004, n = 12). In addition, Bland-Altman analysis yielded similar results. The bias (95% limit of agreements) was 3.14% (−13.31–19.58%) under fluid therapy (n = 12), whereas during baseline measurements (n = 23) a value of 2.98% (−14.07–20.03%) was obtained.

In comparison with iohexol and amikacin, more variation in total PAH body clearance was observed after repeated measurement in the control group, as can be seen in Figure 4C. This resulted in a lower reproducibility (R = 18.14%) than observed for iohexol and amikacin (Table 2). Within the control group, no significant increase in total clearance was observed (*p* = 0.42). Conversely, a significant increase was observed during fluid administration within the treatment group (*p* = 0.046). However, there was not enough statistical evidence to demonstrate that the increase in total body clearance of PAH was larger in the treatment group than in the control group (*p* = 0.19).

Regarding the estimation of renal clearance of PAH, only eight piglets could be included. To evaluate the renal clearance, it is mandatory to collect urine over the entire period where PAH is urinary excreted. When a leakage from the urine collection bag was observed during one of the two renal clearance measurements, the particular piglet was completely excluded from PAH renal clearance calculations. Unfortunately, in only eight out of 23 piglets (4 control and 4 treated), urine collection could be performed twice (M1 and M2) without leakages. No significant differences were observed between the two measurements within the control (*p* = 0.31) and treatment group (*p* = 0.063) and between groups (*p* = 0.44). This observation could be related to the combination of low sample size, the use of a nonparametric statistical test, and the limited reproducibility of the PAH clearance.

Concerning V_ss_, contrasting results were obtained for iohexol, amikacin, and PAH. In case of iohexol, a significant increase in V_ss_ during fluid administration was observed within the treatment group (*p* = 0.008). This increase in V_ss_ was also significantly higher in the treated than the control group (*p* = 0.044). For amikacin on the other hand, a significant decrease in V_ss_ was observed in the control group, when comparing M1 with M2 (*p* = 9.77 × 10^−4^). No significant effect on V_ss_ was observed within the treatment group (*p* = 0.91). For PAH, no significant changes were observed in V_ss_ within the blank group (*p* = 0.97) and between groups (*p* = 0.12). In contrast, the increase in V_ss_ within the treatment group was significant (*p* = 0.021).

Within the treatment group, fluid administration resulted in a significant decrease in AUC_0→inf_ for iohexol (*p* = 0.012) and amikacin (*p* = 0.004). For PAH, the decrease was borderline not significant (*p* = 0.077). In addition, no significant effects in the control group could be observed for iohexol (*p* = 0.70), amikacin (*p* = 0.97), and PAH (*p* = 0.97). This resulted in a significant different change in AUC_0→inf_ when comparing the control and the treatment group for amikacin (*p* = 0.002). For iohexol, the change in AUC_0→inf_ was borderline not statistically significant (*p* = 0.051). In case of PAH, no significant different change was observed when comparing both groups (*p* = 0.21).

### Effect of Fluid Administration on Urine Output and Hematocrit

**FIGURE 6:**
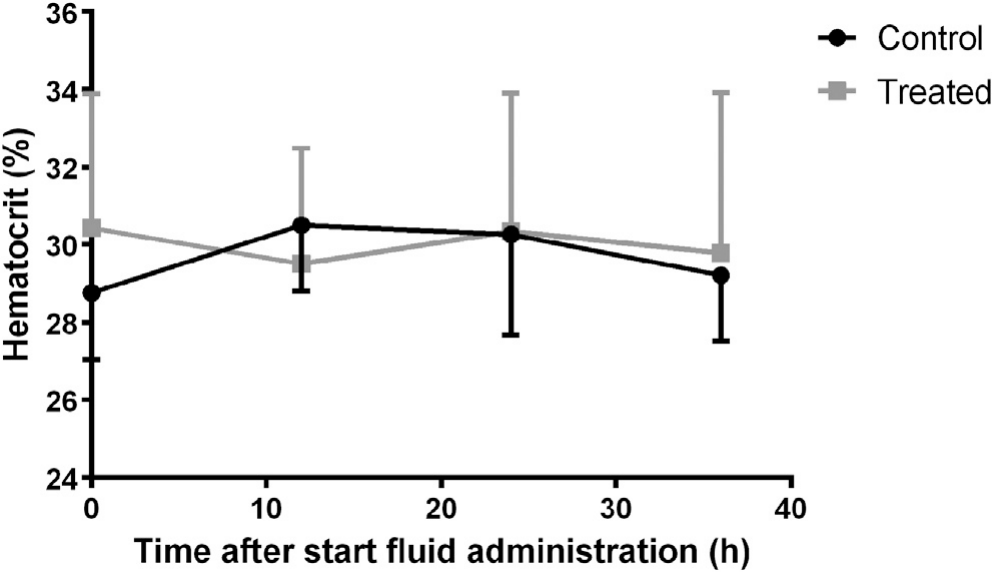
Variation of hematocrit over time in the control (n = 11) and fluid treated (n = 12) group. Mean±SD are presented.

Changes in urine output were evaluated in piglets with twice a complete urine collection. The median (25th percentile-75th percentile) urine output at baseline measurement was 1.12 (1.08–1.15) and 1.45 (1.25–1.66) mL/kg/h in the control (n = 4) and treatment group (n = 4), respectively. In the control group, the urine output stayed quite constant with a median value of 1.12 (1.06–1.32) mL/kg/h at second measurement. During fluid administration the urine output in the treated piglets rose to 4.19 (3.95–4.23) mL/kg/h, which corresponded with an average increase of 185% (range +111% to +241%) against baseline measurement. Consequently, a significant higher increase in urine output was observed at second measurement in the treatment group compared with the control group (*p* = 0.029). In Figure 6 the change in hematocrit values over time is illustrated. No statistical effect of time was observed on hematocrit within each group. However, the percentage change in plasma volume, calculated by the formula of van Beaumont, differed statistically between groups (*p* = 0.006). Statistical analysis revealed differences between the groups at 12 (*p* < 0.001) and 24 h (*p* = 0.02) after the start of fluid administration. This was primarily caused by a percentage decrease of plasma volume in the control group (median %∆P_12h_: -6.70; median %∆P_24h_:−4.55) rather than an increase in plasma volume in the treated pigs (median %∆P_12h_: 2.99; median %∆P_24h_:2.53). No significant difference was observed after 36 h (*p* = 0.31).

## Discussion

To the authors’ knowledge, this is the first study evaluating the effect of prolonged (36 h), continuous fluid administration on the renal function in pigs. The rate of saline administration in this study was based on the porcine water requirements and the daily amount of IV fluids given to critically ill children admitted to the intensive care unit (ICU). Since maintenance IV fluid administration to piglets is no common practice, the rate of fluid administration during this trial was primarily based on the oral fluid intake of piglets. Reported porcine daily water intake is highly variable with values ranging from 1,757 to 2,580 mL for piglets within the 10–20 kg BW category. This resulted in values, indexed for BW, of approximately 4.2–7.7 mL/kg/h (Brooks et al., 1984; Bigelow and Houpt, 1988; SCS Boehringer Ingelheim Comm. V., 2018). Compared to children within the same weight category, the fluid requirements of piglets seem slightly higher. In children, routine maintenance IV fluid rates are generally determined by the Holliday–Segar formula (Holliday and Segar, 1957). For infants weighing 3.5–10 and 11–20 kg the daily fluid requirement is 100 mL/kg or 4.2 mL/kg/h and 1,000 mL plus an additional 50 mL/kg for every kg over 10 kg BW, respectively. In case of critical illness, fluid requirements exceed the routine maintenance requirements. In the study of Van Der Heggen *et al.*, the amount of IV fluids administered to critically ill pediatric patients ranged between 3.4 and 6 mL/kg/h (interquartile range) (Van der Heggen et al., 2019). Since the aim of this study was to evaluate the potential effect of fluid administration on renal function, a fluid infusion rate of 6 mL/kg/h, which probably slightly overestimates the normal fluid requirements in piglets, was selected for this study. At this rate, the volume of administered fluid is approximately 1.5 times greater than the routine maintenance fluid requirement in children and it approximates the volumes administrated to critically ill children. In addition, at 6 mL/kg/h the administered volume is roughly 1.5 times the reported lower limit of the normal porcine daily water intake. The latter was also substantiated when taking the porcine water balances reported by Mroz *et al.* into account*.* An increase in urinary output from a baseline value of 1.46 to 3.99 mL/kg/h, as on average observed in this study, is related with an increase in (oral) fluid intake from 3.65 to 6.23 mL/kg/h under the assumption that the change in water consumption is exclusively matched by changes in urine output (Mroz et al., 1995; Patience, 2012). The underlying calculations are presented in the Supplementary Material. This observation confirms that the normal oral fluid requirements in the studied piglets were exceeded by a factor of roughly 1.7 during IV fluid administration. Evaluation of the normal oral fluid intake of the piglets would have had an added value to determine the degree of overestimation by fluid therapy. However, the stable infrastructure did not allow accurate measurement of this parameter.

The constant rate infusion of 0.9% saline at a rate of 6 mL/kg/h had a significant effect on the clearance of the administered drugs. The GFR, measured as the total iohexol clearance, increased with on average 15%. These results confirm that IV fluid administration indeed can contribute to the development of ARC. However, in hospitalized patients, volume of administered fluid was not always identified as risk factor for developing ARC (Campassi et al., 2014; Declercq et al., 2016; Hirai et al., 2016; Van der Heggen et al., 2019). A possible explanation for this discrepancy could be poor recording of fluid balances in ARC studies by, for example, not taking into account oral fluid intake or IV fluids concomitantly administered with IV drugs. Especially in patients admitted to the intensive care unit considerable amounts of fluid are given concomitantly with the various administered drugs. Also, the large interindividual differences in response to fluid administration can be responsible for the lack of statistical association between fluid administration and ARC in previous studies. Notwithstanding the presented study was conducted in relatively strict controlled conditions using healthy individuals, large interindividual differences in response to fluid therapy were noted in the studied pigs. Similar or even larger interindividual variation might be expected in the (critically) ill pediatric patient population as fluid requirements can be significantly altered due to the underlying disease state.

Likewise for iohexol, a similar increase in total body clearance was observed for amikacin. Amikacin is an antibiotic almost entirely eliminated by glomerular filtration and has been previously used to quantify developmental changes in GFR in neonates (De Cock et al., 2012; Zhao et al., 2013). In this study, amikacin demonstrated a good correlation and agreement with the measured GFR, determined as iohexol total body clearance. This indicates that amikacin clearance can be used to quite accurately evaluate changes in GFR. Furthermore, a high reproducibility of iohexol and amikacin total body clearance measurements was observed in the control group, indicating clearances of both compounds are suitable to evaluate GFR changes in pigs. In human and dog studies, a similar reproducibility for iohexol clearances was obtained (Arvidsson and Hedman, 1990; Gaspari et al., 1998; Finco et al., 2001). The reproducibility of PAH total body clearance was much lower. This was partially attributed to two extreme increases in CL_TOT_ of PAH at second measurement in the control group, as illustrated in Figure 4C. Additionally, the control piglet demonstrating the highest increase in PAH clearance corresponds to the piglet with the highest deviation during repeated iohexol clearance determination. As a consequence, there was a lack of statistical evidence to demonstrate that the increase in total PAH clearance was higher in the treatment group compared to the control group. Nevertheless, a significant effect of fluid administration on the total PAH clearance was observed within the treatment group (*p* = 0.046). Since the two control piglets with an extreme increase in CL_TOT_ of PAH were also included in the calculations for renal PAH clearances, no significant effect on this parameter was observed. Nevertheless, in the treatment group individual values of CL_R_ of PAH increased with 33–49% from baseline value after fluid administration. Yet, this observation should be interpreted with care, since too little information is available to be conclusive about the reproducibility of ERPF determinations.

Numerous drugs are mainly excreted by the kidney and therefore their PK can be affected by ARC. In this study, amikacin was selected as model drug to evaluate the effect of fluid administration on the PK. Besides the total clearance, also the AUC_0→inf_ of amikacin was significantly affected by fluid administration. Consequently, the clinical efficacy of this compound can be altered. Indeed, the AUC/MIC ratio is proposed to be an indicator of bacterial killing and clinical efficacy of aminoglycosides (Bland et al., 2018). In concordance with that, also other antibiotics with AUC/MIC as PK/PD indicator of clinical efficacy can be negatively affected by extensive fluid therapy. Though it is important to mention that, in concordance with amikacin and iohexol total body clearances, large interindividual variation in effect of fluid administration on AUC_0→inf_ was observed. Whereas for some pigs amikacin AUC_0→inf_ was reduced with 20–30% under influence of fluid therapy, in other pigs, receiving equal fluid rates, no or negligible effects on drug exposure were noticed. Since the effect of fluid administration on drug exposure is not consistent, the treatment implication varies between patients. Therapeutic drug monitoring would allow the identification of patients negatively impacted by fluid administration.

No significant change in hematocrit over time was observed in either the treatment (*p* = 0.91) or the control group (*p* = 0.69), indicating no major plasma expansion was present. In contrast, a decrease in hematocrit was previously observed in case of IV fluid administration as bolus (Crawford and Ludemann, 1951a). In the presented study, a significant difference in change in plasma volume between the groups was observed at 12 and 24 h after the start of the fluid administration. This effect rather contributed to a decrease in plasma volume in the control group than an increase in the treatment group. An explanation for this observation is lacking but is in accordance with the observed decline in V_ss_ of amikacin in the control group. In contrast to amikacin, iohexol showed a slight but significant increase in V_ss_ after fluid administration (*p* = 0.008). Iohexol has previously been used as marker for the assessment of the extracellular fluid (ECF) volume (Zdolsek et al., 2005; Finch et al., 2015). The volume of distribution of iohexol is hereby used as an indicator of the ECF volume. Based on the changes in V_ss_ indexed for body weight, it can be concluded that there was a significant increase in ECF volume within the treatment group. Nevertheless, this increase was limited to a median value of 8% (+2 – +17%). Within the control group the change in ECF was negligible [0% (−3 – +2%)].

Generally, it is believed that the GFR is stable or will increase following plasma volume expansion with saline (Burg, 1981; Stenvinkel et al., 1992). Yet, studies evaluating the renal effects of IV administered fluids in children are scare. To the authors’ knowledge, only Leake *et al.* evaluated the renal response to intravenous infusions in pediatric patients (Leake et al., 1976). Also in adults, limited studies are available about the implications of IV fluid administration on the renal function. Most of these studies evaluate the effect of IV bolus administered saline on the glomerular response (Crawford and Ludemann, 1951b; Stenvinkel et al., 1992; Hahn et al., 2016). Between these studies, highly variable results are reported. According to Smith *et al.* the human variability and limited response to IV bolus saline is species-specific, since more pronounced and uniform effects are observed in dogs and rats. This could be attributed to a greater glomerular stability in man than dog/rat or the presence of currently unidentified factors in its regulation that are less important for other species (Smith, 1964). In addition, other factors, such as age and disease state, contribute most likely to the reported variability in human renal response to fluid administration. In contrast to bolus IV fluid administration, studies evaluating in-depth the renal effects of long-term continuously administered fluids in both children and adults are currently lacking. The results, presented in this porcine study, indicate that continuously administered fluids may contribute to the development of ARC. Nevertheless, it is mandatory to confirm this effect in the human population. In addition, further research is necessary to determine the contribution of fluid therapy to the development ARC under (critically) ill conditions.

This study had some limitations. First, the white blood cell count and formula in combination with fever were used to evaluate the presence of infection/inflammation. These evaluation criteria might have a limited sensitivity and specificity in the detection of infection/inflammation in the studied pigs. Secondly, only one type of fluid (0.9% saline) and administration rate (6 mL/kg/h) was evaluated. The response to other commonly applied IV fluid types and other rates may differ from the one observed in this study.

## Conclusion

In conclusion, both the total body clearance of iohexol, which is a measure of the GFR, and amikacin clearance were increased during the CRI of 0.9% saline at 6 mL/kg/h over 36 h, indicating a possible contribution of fluid therapy to the development of ARC. Furthermore, amikacin clearance showed good agreement and correlation with iohexol clearance. Consequently, this compound can be used to evaluate the GFR. Due to the increased clearance, the AUC_0→inf_ of amikacin decreased significantly, which illustrates the potential impact of fluid administration on drug PK and potential drug efficacy. Further research is necessary to confirm these results in humans.

## Data Availability Statement

The raw data supporting the conclusions of this article will be made available by the authors, without undue reservation.

## Ethics Statement

The animal study was reviewed and approved by the Ethical Committee of the Faculty of Veterinary Medicine and the Faculty of Bioscience Engineering of Ghent University (EC 2017/24).

## Author Contributions

LD, MD, SC, PP, and PC contributed to conception and design of the study. LD performed and coordinated the animal trial, performed the bioanalytical and pharmacokinetic analysis, aided in the statistical analysis, and drafted the manuscript. KG performed the statistical analysis. MD aided in the pharmacokinetic analysis. All authors contributed to manuscript revision and read and approved the submitted version.

## Funding

This study was supported by the Special Research Fund of Ghent University (Grant No. BOF16/DOC/285).

## Conflict of Interest

The authors declare that the research was conducted in the absence of any commercial or financial relationships that could be construed as a potential conflict of interest.
