# Supplementary material for: Unraveling the Contribution of Fluid Therapy to the Development of Augmented Renal Clearance in a Piglet Model
Source: Front Pharmacol. 2021 Jan 26;11:607101. doi: 10.3389/fphar.2020.607101 (PMC7870502; doi:10.3389/fphar.2020.607101)
Supplement: Supplementary file 1 [file datasheet1.pdf]

Supplementary Material

1 Bio-analysis of amikacin in plasma

Plasma concentrations of amikacin were quantified using an in-house validated ultra-high-performance liquid chromatography-tandem mass spectrometry method (UHPLC-MS/MS). The method was validated based on European and international guidelines and recommendations (Knecht and Stork, 1974; European Commission, 2002; Committee for Medicinal Products for Veterinary Use, 2015). The validation encompassed an evaluation of the following characteristics: linearity using matrix-matched calibrator samples (correlation coefficient (r) and goodness-of-fit coefficient (gof)), within-run and between-run precision and accuracy, lower limit of quantification (LLOQ), limit of detection (LOD) and carry-over.

A stock solution (SS) of 1 mg/mL amikacin (amikacin sulphate, Santa Cruz Biotechnology, Dallas, Texas, United States) in 1% formic acid (FA) in ultrapure (UP) water (H_2O) was made and stored at 2-8°C. Working solutions (WS) of amikacin were prepared by appropriate dilution of the SS with 1% FA in UP H_2O. A 1 mg/mL SS of the internal standard (IS), tobramycin (tobramycin sulphate, Sigma-Aldrich, Bornem, Belgium), was prepared in water and stored at ≤ -15°C. This solution was diluted with UP H_2O to obtain a WS of 10 µg/mL, which was stored at 2-8°C.

Sample preparation consisted of sequential addition of 50 µL of 1% FA in H_2O and 50 µL of IS WS to 100 µL of plasma. After vortex mixing (15 s), 50 µL of 10% trichloroacetic acid (Merck, Darmstadt, Germany) in H_2O was added to each sample. Subsequently, samples were vortex mixed for 15 s and centrifuged for 10 min (16,200 × *g*, 4°C). The obtained supernatant was filtered through a Millex® nylon syringe filter (0.22 µm) and transferred into an autosampler vial. A 10 µL aliquot of the final solution was injected onto the UHPLC-MS/MS instrument.

Chromatographic separation was achieved on a BEH C_{18}-column (50 mm x 2.1 mm internal diameter, 1.7 µm) in combination with a guard column of the same type, both from Waters (Zellik, Belgium). Mobile phase A consisted of 5 mM pentafluoro propionic acid (PFPA, Sigma-Aldrich, Bornem, Belgium) in H_2O:acetonitrile (50:50,V:V). As mobile phase B, 5 mM PFPA in H_2O was used. The following gradient elution program was run: start condition (10% A, 90% B), 0.0–3.0 min (linear gradient to 60% B), 3.0–5.0 min (linear gradient to 10% B), 5.0–6.0 min (90% A, 10% B), 6.0-6.1 min (linear gradient to 90% B) and 6.1-10.0 (10% A, 90% B). Flow rate was set at 300 µL/min.

Mass spectrometric detection was performed on a Waters Quattro Premier equipped with a heated electrospray ionization (h-ESI) probe operating in the positive ionization mode. The following transitions (*m/z*) were used for identification and quantification, respectively, for amikacin: 586.3 > 425.4 and 586.3 > 163.2 and for tobramycin: 468.2 > 324.3 and 468.2 > 163.2.

Matrix-matched calibration curves were prepared over a concentration range of 0.5-20 µg/mL. The calibration model was quadratic with $1/x^2$ weighting. When sample concentrations above the validated concentration range were observed/expected, analysis was performed on a reduced sample volume (50 µL) supplemented with 50 µL blank porcine plasma.

In Table S1 an overview of the validation results is presented. The LLOQ was 0.5 µg/mL. The LOD, defined as the lowest concentration which could be recognized by the detector with a signal-to-noise (S/N) ratio of ≥3, was 22.98 ng/mL. No carry-over was observed in the solvent sample injected after the highest calibrator.

Table S1: Validation results of the evaluation of calibration (correlation coefficient (r) and goodness-of-fit coefficient (gof)), lower limit of quantification (LLOQ), limit of detection (LOD), and within-day and between-day accuracy and precision for quantification of amikacin in porcine plasma.

**Concentration range (µg/mL)**	**r**	**gof (%)**	**LLOQ (µg/mL)**	**LOD (µg/mL)**
0.5-20	0.998-0.999	3.56-6.30	0.50	0.023
	**Within-day (n=6)**		**Between-day (n=3 x 6)**	
**QC-level**	**Accuracy (%)**	**Precision (%RSD)**	**Accuracy (%)**	**Precision (%RSD)**
**0.5 µg/mL**	-3.5	8.4	4.5	12.8
**5 µg/mL**	2.9	4.4	2.9	4.1
**17.5 µg/mL**	-2.5	2.3	-2.0	3.5

Acceptance criteria for linearity: r > 0.99 and gof < 10%

Acceptance criteria for within-and between-day accuracy: -20 to +10%

Acceptance criteria for within-day precision: 10%

Acceptance criteria for between-day precision: 0.5 µg/mL: 17.8%; 5 µg/mL: 12.6%; 17.5 µg/mL: 10.4%

2 Bio-analysis of para-aminohippuric acid in urine

For the analysis of PAH in urine, the same UHPLC method was used as described by Dhondt et al (2019) (Dhondt et al., 2019). Sample preparation consisted of the addition of 12.5 µL of 1 M hydrochloric acid and 25 µL of H_2O to 50 µL of urine. After vortex-mixing, the samples were equilibrated 10 min at room temperature. Next, 1,912.5 µL of H_2O was added to the samples. After vortex-mixing, 250 µL of the sample was added together with 750 µL of H_2O and 12.5 µL of IS solution (5 µg/mL $^{13}C_6$-PAH) in a vial. An aliquot of 3 µL of the final solution was injected on the column. Samples expected to have concentration levels higher than the highest calibrator were diluted 1/10 with H_2O. Subsequently, 50 µL of this solution underwent the same sample treatment as the non-diluted samples. LLOQ and LOD were 0.25 µg/mL and 7.6 ng/mL, respectively. The method was validated based on European and international guidelines and recommendations (Knecht and Stork, 1974; European Commission, 2002; Committee for Medicinal Products for Veterinary Use, 2015). The validation encompassed an evaluation of the following characteristics: linearity using matrix-matched

calibrator samples (correlation coefficient (r) and goodness-of-fit coefficient (gof)), within-run and between-run precision and accuracy, lower limit of quantification (LLOQ), limit of detection (LOD) and carry-over. Validation results are presented in Table S2.

Table S2: Validation results of the evaluation of linearity (correlation coefficient (r) and goodness-of-fit coefficient (gof)), lower limit of quantification (LLOQ), limit of detection (LOD), and within-day and between-day accuracy and precision for quantification of para-aminohippuric acid in porcine urine.

**Concentration range (µg/mL)**	**r**	**gof (%)**	**LLOQ (µg/mL)**	**LOD (µg/mL)**
0.25-100	0.999	2.91-4.74	0.25	0.0076
	**Within-day (n=6)**		**Between-day (n=3 x 6)**	
**QC-level**	**Accuracy (%)**	**Precision (%RSD)**	**Accuracy (%)**	**Precision (%RSD)**
**0.25 µg/mL**	0.1	9.8	5.4	12.0
**5 µg/mL**	1.0	4.0	0.3	3.6
**50 µg/mL**	-1.6	3.8	-2.0	2.5
**500 µg/mL***	0.3	2.9	0.2	2.5

Acceptance criteria for linearity: r > 0.99 and gof < 10%

Acceptance criteria for within-and between-day accuracy: -20 to + 10%

Acceptance criteria for within-day precision: 10%

Acceptance criteria for between-day precision: 0.25 µg/mL: 19.7%; 5 µg/mL: 12.6%; 50 µg/mL: 8.9%; 500 µg/mL: 6.3%

***** Diluted QC sample

3 Estimation of the extent to which fluid administration at 6 mL/kg/h overestimates the fluid requirements of pigs

3.1 Normal water intake/ ad libitum access to water

From the urine output observed in the studied pigs under *ad libitum* access to water, it is possible to estimate the amount of water that the piglets have drunk by using the porcine water balances (input-output) reported by Mroz *et al.* (cited by Patience *et al.*)(Mroz et al., 1995; Patience, 2012).

Available parameters			***Calculations/Results***		
Output			***Output***		
	**Fraction of output**	**mL/kg/h**		**Fraction of output**	**mL/kg/h**
**Urine**	0.3[a]	**1.46** [b]	**Urine**	0.3	**1.46**
**Growth**	0.14[a]	UNKNOWN	**Growth**	0.14	0.68
**Feces**	0.28[a]	UNKNOWN	**Feces**	0.28	1.36
**Skin-lung**	0.29 [a]	UNKNOWN	**Skin-lung**	0.29	1.41
**Total output**		UNKNOWN	**Total output**		4.87

[a] Values adopted from Mroz *et al.* and Patience *et al.*

[b] Average urine output of the studied pigs under *ad libitum* access to water

Input			***Input***		
	**Fraction of input**	**mL/kg/h**		**Fraction of input**	**mL/kg/h**
**Drinking water**	0.75 [a]	UNKNOWN	**Drinking water**	0.75	**3.65**
**Others**	0.25 [a]	UNKNOWN	**Others**	0.25	1.22
**Total input**		UNKNOWN	**Total input**		4.87

[a] Values adopted from Mroz *et al.* and Patience *et al.*

Following the water balances of Mroz *et al.* an urine output of 1.46 mL/kg/h corresponds with an oral water intake of 3.65 mL/kg/h.

3.2 Fluid administration at 6 mL/kg/h

Similar calculations were performed as under normal water intake/ ad libitum access to water. Nevertheless, it was assumed that the change in water consumption (above normal) was exclusively matched by changes in urine output. Consequently, input via 'others' and output via 'growth', 'feces' and 'skin-lung' remained constant and all equal the values as observed under normal water intake/ *ad libitum* access to water.

Available parameters		***Calculations/Results***	
Output		***Output***	
	**mL/kg/h**		**mL/kg/h**
**Urine**	**3.99**[d]	**Urine**	**3.99**
**Growth**	0.68[c]	**Growth**	0.68
**Feces**	1.36[c]	**Feces**	1.36
**Skin-lung**	1.41[c]	**Skin-lung**	1.41
**Total output**	UNKNOWN	**Total output**	7.45

[c] Assumed to be equal as under *ad libitum* access to water
[d] Average urine output of the studied pigs receiving 6 mL/kg/h normal saline

Input		***Input***	
	**mL/kg/h**		**mL/kg/h**
**Water**	UNKNOWN	**Water**	**6.23**
**Others**	1.22[c]	**Others**	1.22
**Total input**	UNKNOWN	**Total input**	7.45

[c] Assumed to be equal as under *ad libitum* access to water

Using the water balances reported by Mroz *et al.*, an urine output of 3.99 mL/kg/h corresponds with an (oral) water intake of 6.23 mL/kg/h. The latter value approximates the rate of fluid administration (6 mL/kg/h) used in this study. Consequently, this rate overestimated the normal water intake roughly 1.7 (6.23/3.65) times.

4 References

Committee for Medicinal Products for Veterinary Use (2015). "VICH Topic GL49: Studies to Evaluate the Metabolism and Residue Kinetics of Veterinary Drugs in Food Producing Animals: Validation of Analytical Methods Used in Residue Depletion Studies". (London, UK).

Dhondt, L., Croubels, S., De Cock, P., De Paepe, P., De Baere, S., and Devreese, M. (2019). Development and validation of an ultra-high performance liquid chromatography-tandem mass spectrometry method for the simultaneous determination of iohexol, p-aminohippuric acid and creatinine in porcine and broiler chicken plasma. *Journal of Chromatography B-Analytical Technologies in the Biomedical and Life Sciences* 1117**,** 77-85. doi: 10.1016/j.jchromb.2019.04.017.

European Commission (2002). European Commission Decision (EC) No 2002/657/EC Implementing Council Directive 96/23/EC concerning the performance of analytical methods and the interpretation of results. *Official Journal Of The European Communities***,** 8-36.

Knecht, J., and Stork, G. (1974). Prozentuales und logarithmisches Verfahren zur Berechnung von Eichkurven. *Fresenius' Zeitschrift für analytische Chemie* 270(2)**,** 97-99. doi: https://doi.org/10.1007/BF00434058.

Mroz, Z., Jongbloed, A.W., Lenis, N.P., and Vreman, K. (1995). Water in pig nutrition: physiology, allowances and environmental implications. *Nutrition Research Reviews* 8(1)**,** 137-164. doi: 10.1079/NRR19950010.

Patience, J.F. (2012). The importance of water in pork production. *Animal frontiers* 2(2)**,** 28-35. doi: https://doi.org/10.2527/af.2012-0037.
